# Human lesions and animal studies link the claustrum to perception, salience, sleep and pain

**DOI:** 10.1093/brain/awac114

**Published:** 2022-03-28

**Authors:** Huriye Atilgan, Max Doody, David K. Oliver, Thomas M. McGrath, Andrew M. Shelton, Irene Echeverria-Altuna, Irene Tracey, Vladyslav V. Vyazovskiy, Sanjay G. Manohar, Adam M. Packer

**Affiliations:** 1 Department of Physiology, Anatomy, and Genetics, University of Oxford, Oxford OX1 3PT, UK; 2 Department of Experimental Psychology, University of Oxford, Oxford OX2 6GG, UK; 3 Wellcome Centre for Integrative Neuroimaging, FMRIB Centre, Nuffield Department of Clinical Neurosciences, John Radcliffe Hospital and Merton College, University of Oxford, Oxford OX3 9DU, UK; 4 Nuffield Department of Clinical Neurosciences, John Radcliffe Hospital, University of Oxford, Oxford OX3 9DU, UK

**Keywords:** claustrum, lesion, perception‌, salience, sleep, pain

## Abstract

The claustrum is the most densely interconnected region in the human brain. Despite the accumulating data from clinical and experimental studies, the functional role of the claustrum remains unknown. Here, we systematically review claustrum lesion studies and discuss their functional implications. Claustral lesions are associated with an array of signs and symptoms, including changes in cognitive, perceptual and motor abilities; electrical activity; mental state; and sleep. The wide range of symptoms observed following claustral lesions do not provide compelling evidence to support prominent current theories of claustrum function such as multisensory integration or salience computation. Conversely, the lesions studies support the hypothesis that the claustrum regulates cortical excitability. We argue that the claustrum is connected to, or part of, multiple brain networks that perform both fundamental and higher cognitive functions. As a multifunctional node in numerous networks, this may explain the manifold effects of claustrum damage on brain and behaviour.

## Teaser

The claustrum is the most densely interconnected region in the human brain. By reviewing the literature on human lesions of the claustrum and linking it to the recent explosion in animal research, Atilgan *et al*. argue that the claustrum contributes to perception, pain, sleep and salience via its integral roles in different networks.

## Introduction

**Figure 1:**
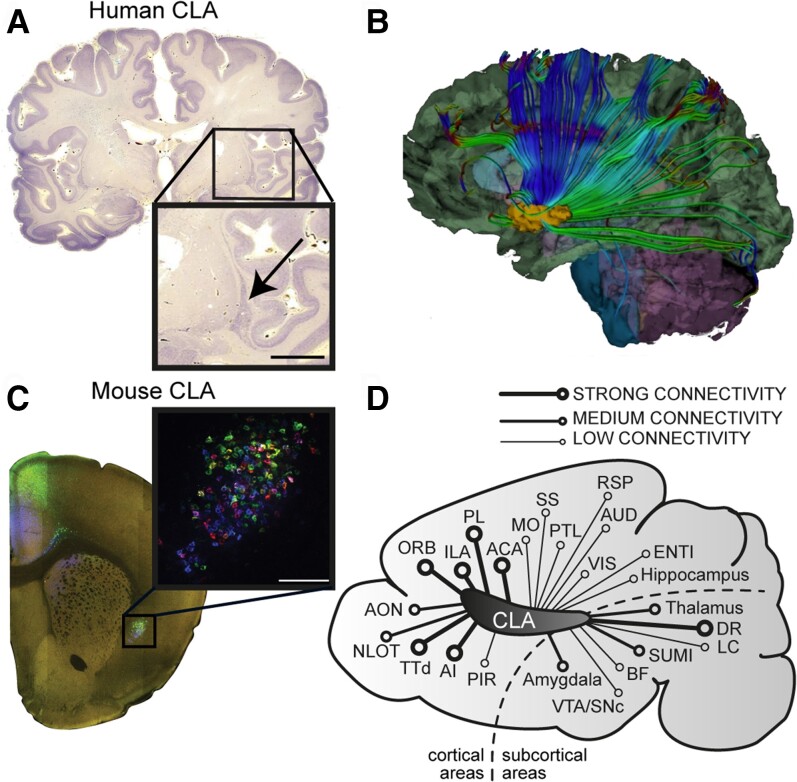
**The location and connectivity of the claustrum.** (**A**) Nissl stained human coronal brain section with *inset* showing the location of the claustrum. (**B**) White matter tractography image showing outgoing connections from the claustrum reprinted from Torgerson *et al*.^17^ with permission from John Wiley and Sons ©2015. (**C**) Mouse coronal brain section with inset showing the claustrum labelled by multi-colour retrograde tracers. (**D**) Schematic illustration of a sagittal mouse brain section showing the relative connectivity strength between the claustrum and other brain regions. Connectivity strength was assessed based on the data provided in Zingg *et al*.^15^ and Wang *et al*.^12^ Most but not all connections are reciprocal (see reviews Mathur^26^ and Jackson *et al*.^25^). ACA = anterior cingulate area; AI = anterior insular cortex; Aud = auditory cortex; AON = anterior olfactory nucleus; BF = basal forebrain; CLA = claustrum; DR = dorsal raphe; ENTI = entorhinal cortex; ILA = infralimbic area; LC = locus coeruleus; MO = motor cortex; LOT = the nucleus of the lateral olfactory tract; ORB = orbital area; PL = prelimbic area; PTL = parietal cortex; PIR = piriform area; RSP = retrosplenial cortex; SUMI = supramammillary; SS = somatosensory cortex; TTd = dorsal taenia tecta; VTA/SNc = ventral tegmental area/substantia nigra; VIS = visual cortex.

The claustrum is a sheet-like bilateral brain region tucked beneath the insular cortex (Fig. 1). Extensive anatomical work across different species, including humans, monkeys, rodents, reptiles, rabbits, and cats, indicates widespread connectivity between the claustrum and the neocortex,^1–15^ and highlights the need for a more thorough investigation of the claustrum’s circuitry. Strong reciprocal connectivity with most neocortical areas is perhaps the most noteworthy feature of the claustrum,^2,7,9,10,14–16^ which has the highest connectivity in the human brain by regional volume.^17^ Recent work has shown corticoclaustral and claustrocortical connections evoke feedforward inhibition in mice,^6,18^ similar to previous results in cats.^19–22^ While the anatomy, physiology, and putative functions of the claustrum have been reviewed elsewhere,^4,16,23–27^ this review seeks to untangle and shed new light on the functional role of the claustrum.

Understanding the anatomy and connectivity of a brain region can provide important insights into its function. Attempts to find classic structure-function relationships are challenging in highly connected brain regions where many functions are possible due to multiple co-existing pathways. In the case of the claustrum, such widespread connectivity has led to a plethora of hypotheses regarding its function. The claustrum has been implicated in processes including the amplification of cortical oscillations,^28,29^ attentional allocation,^2,4,26,30^ consciousness,^16,31^ cognition,^6^ spatial navigation,^32^ the transfer of information across sensory modalities,^33^ sexual function,^34^ and aesthetic judgement.^35^

In addition to investigating its anatomy and connectivity, examining the consequences of damage to a brain region may provide complementary insights into its function. The lesion approach, including both acute and chronic interventions, has been immensely powerful in localizing language, episodic memory, and even aspects of executive function, in both human and animal studies.^36,37^ While imperfect, loss-of-function approaches can enable subsequent neural activity recordings to focus on stimuli or behaviours implicated by the lost functions. Moreover, lesion studies can help reveal a region’s functional connectivity. However, the claustrum’s contorted shape and bilateral location deep in the brain renders this approach difficult, though some studies have attempted to lesion the claustrum in animals.^38–40^

While the claustrum’s anatomy renders lesion studies challenging, examples of claustrum lesions exist within the literature. To our knowledge no review has comprehensively assessed clinical studies of human claustrum lesions. In this review, we present a meta-analysis of all studies to date that describe cases of claustrum lesions in human patients. We show that claustrum lesions lead to a wide range of signs and symptoms. Most commonly, patients experienced impairments in different levels of cognitive, perceptual, and motor abilities, as well as seizures and disturbances to mental state and sleep. Using these findings, in conjunction with animal studies, we consolidate evidence concerning the claustrum’s role in sensory perception, sleep, pain, and salience, and facilitate a critical re-evaluation of existing hypotheses of the claustrum’s function. Our results do not support prominent hypothesized functions such as multisensory integration and salience processing, but may instead provide evidence for a role in regulating cortical excitability. We hypothesize that the claustrum is connected to, or is a part of, many different networks, and propose that the brain-wide connectivity of the claustrum may have provided a useful scaffold onto which many functional roles could subsequently be built. This may explain the apparent role of the claustrum in fundamental functions such as sleep, as well as in more complex functions such as attention and salience. We argue that the claustrum is likely a multifunctional area, or perhaps possesses a global function to regulate neural activity dynamics across the cerebral mantle. Under this schema, damage to the claustrum would disrupt diverse neurological and behavioural processes.

## Diverse findings following lesion or stimulation of the human claustrum

### Literature search

The potential functions of the claustrum have not been comprehensively analysed by examining human lesion studies because of the relative scarcity of claustral lesions (the only reported work focused specifically on delusional states).^27^ To tackle this, we examined claustrum lesion cases by searching the following terms on PubMed and Scopus: ‘claustrum AND (lesion OR contusion OR injury OR trauma)’ (*n* = 103). Studies were then screened for cases in which reported lesions included the claustrum according to neuroimaging. Thirty-eight individual cases (Supplementary Tables 1 and 2) and 14 cohort studies (Supplementary Table 3) were included in this review. Details of these studies can be found in the Supplementary material.

### Specific claustrum lesions

**Figure 2:**
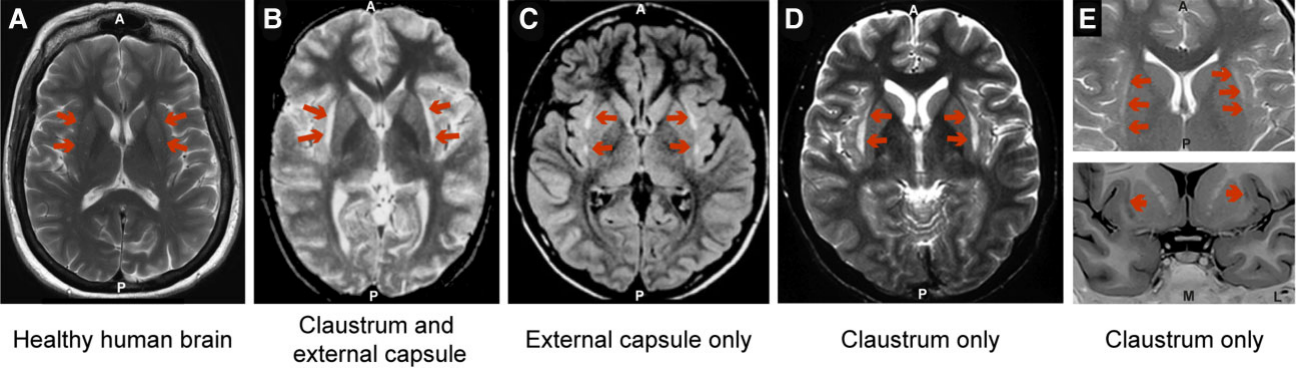
**Similar lesions have been described as affecting either the claustrum, the external capsule, or both.** (**A**) Representative T_2_-weighted image of a healthy human brain. (**B**) T_2_-weighted image showing a lesion reported as affecting the claustrum and external capsule. Reprinted from Sperner *et al*.^45^ with permission from Springer Nature ©1996. (**C**) Fluid-attenuated inversion recovery (FLAIR) image showing a lesion reported as affecting the external capsule. Reprinted from Mumoli *et al*.^43^ with permission from Springer Nature ©2014. (**D**) T_2_-weighted image showing a lesion reported as affecting the claustrum. Reprinted from Ishii *et al*.^42^ with permission from American Society of Neuroradiology ©2011. (**E**) T_2_-weighted image (*top*) and T_1_-weighted image (*bottom*) showing a lesion reported as affecting the claustrum. Reprinted from Silva *et al*.^44^ with permission from Springer Nature ©2018.

Due to the claustrum’s nestled location, vascularization, and morphology, isolated claustrum lesions are rare, and predominantly occur in the spectrum of autoimmune encephalitis. Unfortunately, available clinical imaging techniques lack sufficient resolution to conclusively resolve the claustrum from the external capsule. Studies that reported lesions to the claustrum and external capsule (Fig. 2B), the external capsule only (Fig. 2C), and the claustrum only (Fig. 2D) reveal strikingly similar imaging results. Given this diversity in the reporting of lesions, it is difficult to distinguish truly claustrum specific lesions from those which may impact both the claustrum and adjacent white matter. Disambiguating what caused the lesion can help. For example, autoimmune encephalitis might be caused by anti-neuronal surface antibodies that have a predilection to epitopes on the claustrum, such as voltage-gated potassium channels antibodies and anti-glutamic acid decarboxylase antibody.^41^ On the other hand, pathologies that affect white matter such as vasogenic oedema and demyelination might have a predilection for external and extreme capsules. Thus, the diagnosis offers an important clue to claustrum involvement, despite the imaging in these cases being identical. Nonetheless, the lack of isolated claustrum lesions means lesion studies are inherently problematic. It is therefore difficult to attribute a particular functional role to the claustrum based on lesions alone.

Despite this difficulty, seven case studies have been identified which report relatively exclusive lesions in the claustrum with or without involvement of the external capsule (Supplementary Table 1). Four of these patients presented with status epilepticus and were found to have bilateral lesions affecting the claustrum and external capsule. The four patients also had EEG abnormalities along with a variety of other symptoms including seizures, motor impairment, cognitive impairment, psychotic symptoms, and tremor.^42–45^ Another patient with abnormalities in the right claustrum and bilateral external capsule experienced non-epileptiform EEG abnormalities, along with motor and cognitive impairment.^46^ A 55-year-old patient with an isolated left claustrum lesion following an ischaemic stroke experienced unilateral paraesthesia of the face, tongue, and head—similar to a lacunar syndrome—along with polymodal gustatory, auditory, vestibular, and visual disturbances.^47^ Recently, a patient admitted to hospital with Covid-19 showed bilateral lesions to the claustrum and external capsule. He presented with aggression, cognitive impairment, disorientation, and stupor. Upon follow-up, the claustrum lesions remained despite a complete neuropsychiatric recovery.^48^ For further details of these cases, see Supplementary Table 1.

While the underlying cause of the claustrum damage was not always clear, the various authors suspected a range of aetiologies including viral encephalitis, ischaemic stroke, and seizure induced damage. Despite the similar and isolated brain abnormalities, the patients presented with a wide and inconsistent array of signs and symptoms. The effects reported in these diverse cases are difficult to interpret. They do not offer strong support to any single hypothesis of the claustrum’s function. Conversely, these results suggest that the claustrum may instead have multiple roles, or an, as yet, unidentified global role that impacts multiple behavioural domains. However, further evidence is required to draw any conclusions.

### Non-specific claustrum lesions

Beyond these studies, many others have reported lesions to the claustrum in conjunction with other brain regions (Supplementary Table 2). Other areas commonly included the external capsule (14/38) and insula (12/38), between which the claustrum is interposed, and the hippocampus (9/38). In particular, presumed viral and non-viral encephalitis may cause relatively claustrum-specific changes on MRI neuroimaging, sometimes in conjunction with hippocampal damage. Like the studies with isolated claustrum lesions, these less specific lesion studies also reported a wide variety of symptoms (Table 1). The most common signs and symptoms in individual cases were nonspecific cognitive impairment (19/38), seizures (18/38), motor impairment (17/38), visual disturbances (9/38), non-seizure EEG abnormalities (8/38), loss of consciousness (8/38), speech problems (8/38), auditory disturbances (6/38), sleep disturbances (6/38), delusions (5/38), tremor (5/38), and hallucinations (4/38). We separated these observations into the following four categories: disruption in cognitive, perceptual and motor abilities; changes in electrical activity; changes in mental state; and sleep disturbance.

**Table 1:** Lesioned area, symptoms and seizures reported in case studies with claustrum lesions

**Hemispheric distribution**	Bilateral	22
	Unilateral (Right/Left/Undefined)	16 (9/5/2)
**Extent of lesion**	Claustrum only	7
	External capsule	14
	Insula	12
	Hippocampus	9
	Other cortices	17
	Other subcortices	22
**Signs and symptoms**	Cognitive, perceptual and motor abilities	
	Cognitive impairment	19 (50%)
	Motor disturbance	17 (45%)
	Visual disturbances	9 (24%)
	Speech disturbances	8 (21%)
	Auditory disturbances	6 (16%)
	Tremor	5 (13%)
	Paraesthesia	5 (13%)
	Electrical activity disturbance	
	Seizures	18 (47%)
	Non-seizure EEG abnormalities	8 (21%)
	Mental state	
	Loss of consciousness	8 (21%)
	Hallucinations	4 (11%)
	Delusions (Cotard delusion)	5 (2) (13%)
	Sleep disturbances	6 (16%)
**Seizure type (patients may fit >1 severities)**	1) Partial	7 (18%)
	2) Generalized	15 (39%)
	3) Status epilepticus	13 (34%)
	4) Refractory status epilepticus	10 (26%)

### Claustral lesions impair cognitive, perceptual and motor abilities

Patients with claustrum lesions were frequently reported to have perceptual and cognitive impairments. In many cases, the lesions impacted patients’ ability to perceive sensory input in various ways. For example, a 12-year-old experienced temporary loss of vision and hearing.^45^ Interestingly, another young child experienced permanent bilateral blindness due to damage to the left calcarine cortex and right claustrum, although the most parsimonious explanation for this finding is undetected damage to the right optic radiation.^49^ In some cases, lower perceptual sensitivity,^50^ and tinnitus^51^ were reported. Somatosensory deficits have also been reported.^47,52^ Cases presumed to be driven by encephalitis often presented with deficits in episodic memory,^46,53,54^ working memory, and reasoning or with disorientation in time.^52^ Claustrum damage has even been described as part of neurodegenerative syndromes such as corticobasal degeneration, where it may be accompanied by a disruption in visual attention functions including spatial orientation, optic ataxia,^55^ and cognitive decline in Lewy body dementia.^56^ Left claustrum damage could lead to aphasia when accompanied by left putamen damage.^57^ Despite the varied pathologies, lesion sites, and inconsistent observations across studies, each patient experienced disturbance to some aspect of their cognitive and perceptual abilities. Claustrum lesions led to a wide variety of sensory deficits, suggesting the claustrum may play a functional role in one or more aspects of sensory processing. Notably, no evidence for specific deficits in multisensory integration was reported.

### Claustrum lesions result in an increased incidence of electrical disturbances

Patients with claustral lesions often experienced disturbances to the electrical activity of the brain, involving generalized seizures (15/38) and status epilepticus (5 min of constant seizures without any return to consciousness in between; 13/38). Generalized seizures were more common when the claustrum was damaged bilaterally (10/15 studies). Several studies^45,51^ reported that patients with claustral lesions developed recurrent generalized seizures, with interictal slowing (3–4 Hz) on EEG. Patients who also had claustrum-associated focal seizures reported experiencing delusions and altered mental state (discussed below), which was coupled with pathological EEG slow-wave activity.^58^ Unfortunately, the majority of the case studies reporting generalized seizures do not report EEG recordings. The studies that did include EEG recordings typically reported generalized slow-wave activity present even in the awake state. A few of these also described generalized spikes or sharp waves.^45,59^

Strikingly, several patients with claustrum lesions exhibited a form of severe epilepsy (10/38) known as refractory status epilepticus. Refractory status epilepticus is a subset of these cases that fail to respond to the two frontline pharmacological treatments for seizures (benzodiazepines and one anticonvulsant).^60^ Studies examining new-onset refractory status epilepticus reported lesions in the claustrum as a common pathology, concomitant with slow-wave activity, spikes and sharp waves, and periodic lateralized epileptiform discharges in EEG recordings.^61–63^ Taken together, these studies suggest that claustrum dysfunction increases global synchronization of the electrical activity of the brain, and potentially leads to seizures.^64^ This is consistent with a putative role for the claustrum in regulating cortical excitability.

### Claustral lesions disturb mental state

Several studies noted that medicated and unmedicated patients with claustrum lesions experienced altered mental states, which can be defined as general changes in brain function characterized by confusion, disorientation and disordered perceptions.^65^ These included non-seizure loss of consciousness, confusion, stupor, delusions, hallucinations, as well as other mild fluctuations in mental state. Loss of consciousness, defined here as an interruption in one’s awareness of the self and the environment, was reported in 8 of 38 case studies. Similarly, a study of combat veterans with penetrating traumatic brain injuries reported that patients with lesions to the claustrum showed an increased duration but not frequency of loss of consciousness relative to patients with lesions in other locations.^66^ Recent lesion mapping has uncovered a distributed circuit associated with loss of consciousness, which peaks in the bilateral claustrum.^67^ These findings suggest that the claustrum may be necessary to support some aspects of maintaining one’s level of consciousness. An alternative explanation is that these processes generate ‘selfhood’, the mental representation of a distinct first-person capable of agency, a role which has been associated with the nearby anterior insula^68^ but might be extended to parts of the claustrum.

Altered mental states were reported across many claustrum lesion case studies. While some patients manifested broadly altered mental states including anhedonia, delirium, or confusion,^61,63^ some had narrower neuropsychiatric features such as delusions with paranoid or religious content,^58,69^ visual hallucinations, and abulia.^70^ One patient with claustral dysfunction attributed to COVID-19 exhibited ‘delirious behavior’.^48^ Even more specific features included delusions of jealousy.^71^ The high frequency of visual and auditory hallucinations, confusion, and delusional states has previously been identified and discussed at length by Patru and Reser.^27^ They argue persuasively that the claustrum is involved in the process of generating and filtering delusional beliefs (described below).

Intriguingly, claustrum dysfunction may also be linked to Cotard delusion. Patients experiencing Cotard delusion may believe that they are dead, do not exist, are decaying, or have lost internal organs.^72^ Intriguingly, two patients described above with claustrum lesions experienced symptoms consistent with Cotard delusion.^69,73^ As only a few hundred instances of Cotard delusions are known worldwide, the finding that one confirmed case of Cotard delusion and another patient with highly overlapping symptoms arose from pathology involving the claustrum is highly suggestive of an association and perhaps a disconnection with ‘self’. Although most reported cases of Cotard delusion do not include claustrum lesions^74–76^ the claustrum was the only area of overlap between the two cases described. These examples of altered mental states following claustrum lesions suggest that the claustrum may be required to modulate complex cognitive representations such as beliefs and desires.

### Claustral lesions disturb sleep

Sleep disturbances were commonly observed in patients with claustrum lesions, usually with other symptoms present. Some studies reported hypersomnia along with altered mental state^63,70^ while others reported restlessness along with motor disturbance.^56^ It is unclear whether these sleep disturbances occurred as a direct result of the claustrum lesions themselves, patients’ medications, or damage to other brain structures. Additionally, these studies did not assess the polysomnographic features of the sleep disturbances or provide a systematic investigation of sleep. No details were reported about whether patients had difficulty initiating and/or maintaining sleep. Recently, a genome-wide analysis suggested a link between insomnia and a disruption in claustrum-hypothalamus-striatum circuitry,^77^ pointing to the role of these areas in sleep initiation. Another mechanism by which circuit-level abnormalities might affect a patient’s sleep-wake cycle is through a disturbance in waking resting state functional connectivity in the default mode network.^78^ Although evidence points to an important functional role of the claustrum in sleep control, lesion studies are too limited to provide conclusive evidence.

### Claustral lesions can result in painful sensations

Multiple case studies included patients who reported painful sensations.^61,79–82^ These sensations varied in character and severity. In the 1950s, two separate studies described patients with unilateral lesions to the claustrum, among other regions, who experienced hyperalgesia to hot and/or cold in the contralateral upper limb, together with hyperaesthesia, depression, and suicidal ideation.^80,82^ Pain with hypoaesthesia has also been reported.^81^ Other cases have described dynamic changes, such as painful cramping beginning in the contralateral hand, with Jacksonian march to the whole of the left side,^79^ or discomfort with spreading paraesthesia,^47^ suggesting that pain could relate to heightened excitability rather than neural loss.

Despite these positive findings, caution must be exercised as many painful sensations might more parsimoniously be attributed to causes other than claustral lesions (e.g. myalgia to fever in Choi *et al*.^61^). Claustrum stimulation has also induced pain, with a participant reporting painful sensations ‘like a knife jabbing’ and of ‘burning’ during claustrum stimulation.^83^ This is noteworthy as brain stimulation rarely produces painful sensations, although when it does it has often been reported following stimulation of a region surrounding the posterior insula and claustrum.^84^ While these are isolated examples, numerous studies have hinted at a connection between the claustrum and pain. However, this possibility has received comparatively less attention than other brain areas.

The lesion studies were inconsistent in their reporting of the neurological examination, and most reported just whether sensation was intact. The lack of any specific mention of pain may reflect an incomplete report, rather than constituting evidence that pain was unaffected. However, it does seem clear that there is no obvious neuropathic pain syndrome associated specifically with claustrum lesions, perhaps contrasting with the more distinct sensory syndromes associated with insula damage.^85^ Future studies should specifically test pain in such focal cases, ideally using quantitative methods.

### Human claustrum stimulation

Like lesion studies, brain stimulation studies can provide strong evidence linking a given region to specific functional roles. The usefulness of stimulation studies is, however, limited by the possibility that observed effects result from excitation or inhibition of brain regions downstream of the region being stimulated.^86^ This caveat is particularly noteworthy when considering the claustrum given its widespread connectivity throughout the brain. Additionally, due to the small volume of the claustrum, stimulation will almost certainly result in depolarization of neurons in nearby tissue.

A recent study by Bickel & Parvizi^83^ obtained claustral stimulation data from five patients with pharmacologically resistant epilepsy, whose implanted electrodes partially overlapped with the claustrum. Four of the five patients experienced changes in somatosensation including ‘a strange skin sensation from the face running down to the leg’; ‘a sense of pressure in the mouth’; ‘sensation around the left knee’; ‘hot flashes’; and ‘acid-like sensation in the throat’. However, none of them reported changes in their awareness of the situation and their surroundings. In 2014, Koubeissi *et al*.^31^ reported that a patient with intractable epilepsy suffered a reversible disruption of consciousness after an electrode near the claustrum was simulated. However, a few caveats apply to this finding. First, the electrode was located in the extreme capsule, near, but not within the claustrum. Second, the patient had previously undergone an ipsilateral temporal lobectomy which may have functional implications. Third, the current applied by Koubeissi *et al*.^31^ was greater in both magnitude and duration than in the Bickel and Parvizi^83^ study. This may have caused widespread blanket depolarization of claustrocortical neurons, producing cortical inhibition,^6^ that subsequently disrupted normal information processes.

The signs and symptoms of claustrum dysfunction caused by claustrum lesions and claustrum stimulation vary based on the pathologies and lesion/stimulation sites. Each patient experienced some aspect of disturbance in cognitive function, perceptual abilities, mental state, electrical activity, or sleep. This great diversity of signs and symptoms suggests a structure with a global role affecting diverse cognitive processes. Remarkably, and in stark contrast to this conclusion, patients with well-delineated surgical removal of unilateral claustra for low-grade cerebral glioma had no observed long-term sensorimotor or cognitive impairment.^87^ The functional role of the unilateral claustrum might be efficiently compensated by neural plasticity of the intact claustrum, explaining why these patients did not suffer any permanent cognitive-perceptual impairments. These studies suggest that due to its involvement in multiple functionally compensated dynamic neural networks the claustrum is involved in numerous neural processes including both high-level cognitive processes as well as more fundamental processes. Taken together, these lesion and stimulation studies highlight the need to critically re-evaluate existing theories of the claustrum’s function.

## Re-evaluating the function of the claustrum

The results described above present the most comprehensive survey of claustrum lesions to date. Paradoxically, the most consistent feature of these claustrum lesion studies is their inconsistency. The diverse and overlapping array of clinical pathologies reported in these lesion and stimulation studies suggests that the claustrum may have a far-reaching role. The task of attempting to draw functional conclusions from this rich and varied data is further complicated by limited and inconsistent investigational practices across human studies.

In the sections below we will look more deeply into certain functional hypotheses in light of the lesion studies and propose avenues for future exploration. We will briefly evaluate the claustrum’s involvement in neural correlates of sensory perception and salience as two prominent theories of the claustrum’s function. We then review the claustrum’s involvement in sleep and pain based on recent physiological studies.

### Sensory processing

Lesion studies detailing sensory and perceptual abnormalities are suggestive of a functional role for the claustrum in sensory processing. These observations are consistent with anatomical and physiological findings in basic research. Connectivity studies have shown strong claustrocortical projections to sensory cortices and minimal corticoclaustral input from primary sensory areas.^12,15,88^ Physiological studies have revealed somatotopic responses, retinotopic responses, and direct responses to auditory stimuli within the claustrum.^89–91^ This anatomical connectivity, when considered in conjunction with single neuron recordings, led to the hypothesis that the claustrum is well positioned to play a key role in multisensory integration.^16,28^ However, individual claustrum neurons have been shown to respond predominantly to one sensory modality^92^ without any preference for the properties of the stimulus (i.e. no differences between vocalizations versus environmental sounds^93^).

These contrary findings might be partially resolved by data showing that claustrum neurons responded to changes in the sensory input^93^ and responded to the novelty of stimuli pairs.^94^ This hints that the claustrum does not encode the physical properties of the sensory stimuli but may instead shape sensory perception. This is supported by an early animal study showing that a claustrum lesion impacted the way primary visual cortex encoded a visual feature,^95^ and by more recent theories that the claustrum may reduce noise in sensory cortices.^2,25^

The link to sensory processing is suggested by several lesion patients experiencing hallucinations and other delusions (as reviewed in-depth by Patru and Reser^27^). In that review, the authors argue that the claustrum is poised to act as a common centre for delusional states. They theorize how the claustrum may be involved in common models of delusion such as the glutamate/dopamine model, Κ-opioid receptor model, and models based on deficits in attention, sensory-gating, salience processing, and prediction. It is, however, important to note that these claustrum-related mechanisms of delusion may be directly related to the sensory abnormalities described in the lesion section.

As reported above, claustrum lesions can lead to sensory effects in multiple sensory domains, suggesting that the claustrum may be involved in the modulation of sensory processing. While it is possible that the claustrum plays a role in generating sensory perception, physiological evidence more strongly suggests a nuanced role in shaping the transition from raw sensory input to perceived output as reviewed elsewhere in detail.^26^

### Salience

Recent studies performed in animals have also suggested a role for the claustrum in salience processing. Salience is the property of a stimulus that makes it noticeable. For example, a sudden loud sound carries perceptual salience, whereas the smell of food has motivational salience for a hungry organism. Put another way, salience can be seen as a process of attributing importance to information, which in turn controls information flow in the brain.

**Figure 3:**
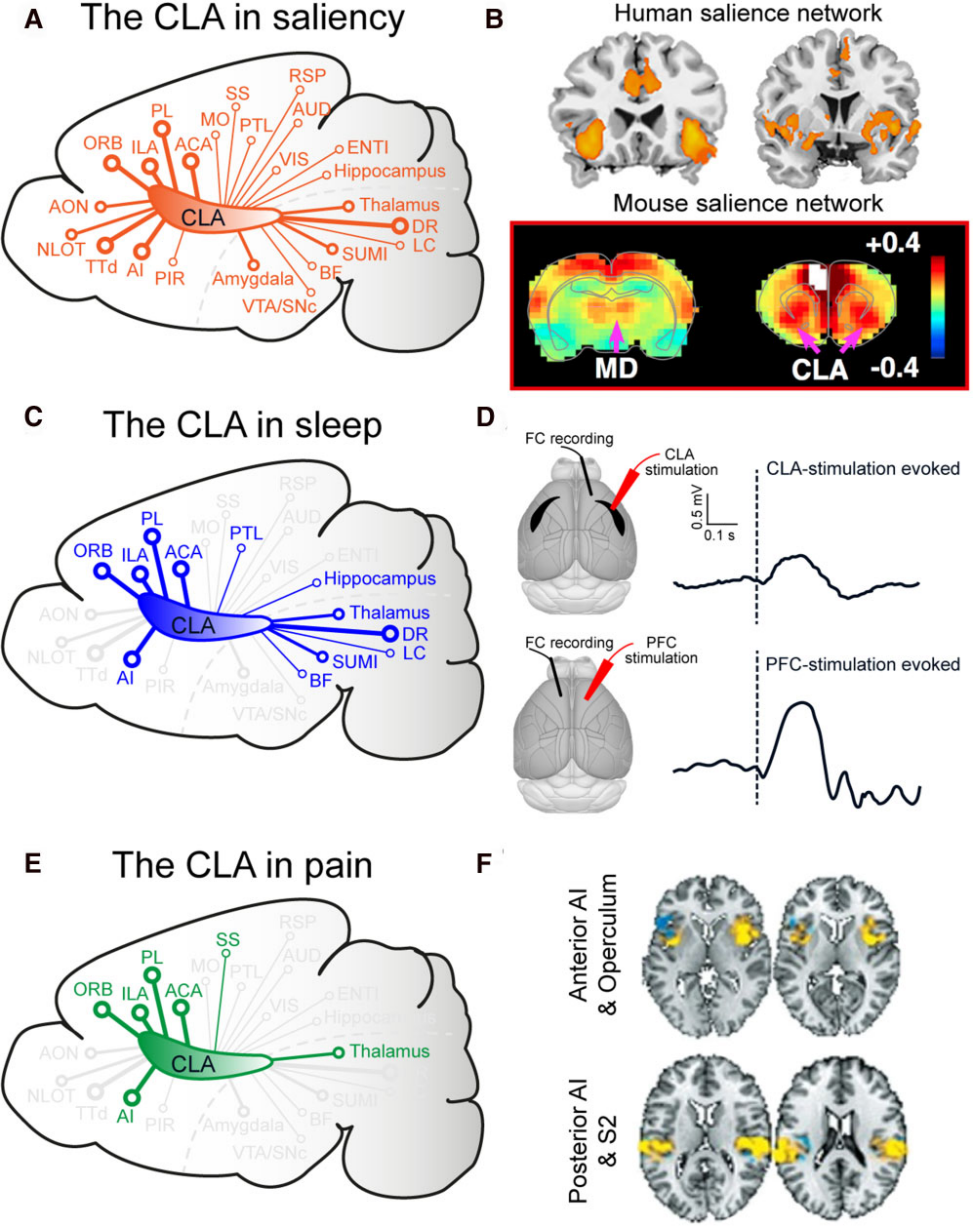
**The claustrum is connected with or part of multiple brain networks.** (**A**) Schematic illustration of a sagittal mouse brain section highlighting the connections between the claustrum (CLA) and regions important in salience. (**B**) Representative functional MRI images showing the human and rodent salience networks adapted from Seeley *et al*.^98^ and Smith *et al*.,^96^ respectively (© Society for Neuroscience). (**C**) Schematic illustration of a sagittal mouse brain section highlighting the connections between the claustrum and regions important in sleep. (**D**) Claustrum and prefrontal cortex (PFC) stimulation both evoke slow-wave like activity. Reprinted from Narikiyo *et al*.^7^ and Vyazovskiy *et al*.,^122^ respectively, with permission from Springer Nature ©2020. (**E**) Schematic illustration of a sagittal mouse brain section highlighting the connections between the claustrum and regions important in pain processing. (**F**) Brain activity to physical pain using multi-study data and machine learning classification tools. Images kindly provided by Tor Wager. FC = frontal cortex; MD = mediodorsal thalamus.

Findings from recent connectivity and physiology studies in animal models support the idea that the claustrum may be part of the ‘salience network.’^6,96^ The salience network is an operationally-defined group of brain regions including the anterior cingulate cortex, insula, amygdala, hypothalamus, ventral striatum, thalamus, and specific brainstem nuclei.^97–99^ Many of these regions are closely connected with the claustrum (Fig. 3A and B). Moreover, the claustrum also receives strong input from regions linked to aversive and rewarding salience through its connections with the amygdala and dopaminergic system, respectively.^100,101^ Recent physiological evidence from rodent models also aligns with these connectivity studies. Firstly, claustrum neurons have been reported to respond to salient sensory stimuli.^23,93^ Secondly, inhibition of the claustrum decreased performance on a forced-choice task only in the presence of a distractor^2^ and regulated the behavioural sensitization to rewarding salient stimuli.^102^ Finally, in a classical conditioning paradigm, claustrum responses most closely correlated with the perceptual salience (which was produced by novelty and unexpectedness) of conditioned and unconditioned stimulus associations.^94^ Based on these and other studies, researchers have proposed a putative role for the claustrum in salience processing.

While much evidence suggests a link, it remains unclear how the claustrum contributes to salience processing. One hypothesis suggests that the salience network is involved with *switching* between the default mode network and the central executive network.^97,99^ This switch allows the brain to deploy the cognitive resources necessary to engage with salient stimuli. Functional MRI studies have suggested that the dorsal anterior insula is linked with this network switch.^97^ However, it is possible that this process may also involve the claustrum. Furthermore, given the connectivity between the claustrum and default mode network, and the inhibitory effect of claustrum stimulation on the cortex, the claustrum is well placed to inhibit the default mode network in response to salient stimuli.^6^

The claustrum is suggested to contribute to salience processing by acting as a coincidence detector. Paired recordings within the claustrum have revealed dense electrical and chemical synapses between interneurons and a relative paucity of excitatory-excitatory connections.^18^ Stimulation of corticoclaustral projections reliably evokes feed-forward inhibition in claustrocortical projection neurons.^18^ This may explain the bimodal activity characteristic of claustrum neuron responses to cortical activation in the presence of feedforward inhibition. As such, strong input within a short temporal window is required to induce suprathreshold responses in the claustrum.^103^ By responding to coincident input from sensory, limbic, executive^104^ and neuromodulatory areas, this coincidence detection architecture could allow the claustrum to signal the salience of stimuli activating multiple neural circuits simultaneously.^29^

On the other hand, the lesion studies discussed above do not support a role for the claustrum in salience. A common sign for the loss of salience in one’s mental representation of the environment is hemispatial neglect.^105^ Hemispatial neglect is a common neuropsychological syndrome, in which patients fail to detect stimuli located contralaterally to a focal hemispheric lesion, even in the absence of primary sensory or motor deficits.^106^ None of unilateral claustrum lesion studies reported spatial hemispatial neglect. Furthermore, claustrocortical projections do not align well with the salience network based on cortico-striatal-thalamic loops. The strong ipsilateral claustrocortical projections do not match with the strong interhemispheric projections between cortical and subcortical areas posited as crucial due to hypotheses about the salience network.^107^ In addition, corticoclaustral projections provide the dominant input to the claustrum (84% of claustrum input originates in the cortex^15^), but salience processing requires substantial non-cortical involvement. While supporting evidence continues to accumulate, questions remain as to whether the claustrum has a role in salience processing.

### Sleep

Many of the lesion studies described above include reports of sleep disturbances. While the studies included limited details of these disruptions, recent developments from animal research support the hypothesis that the claustrum may be involved in sleep (for a more general review of sleep, see Brown *et al*.,^108^ Liu and Dan,^109^ Saper and Fuller^110^ and Vyazovskiy and Harris^111^).

The broad anatomical connectivity of the claustrum makes it well placed to play a role in brain-wide processes such as sleep. Sleep is regulated by numerous brain regions, many of which are strongly connected to the claustrum, including those that are highlighted in Fig. 3C. Of particular relevance to sleep are the enriched serotonin receptors in the claustrum^112–115^ and extensive unidirectional inputs from serotonergic raphe neurons,^7,15^ which are involved in sleep-wake control.^116^ Additionally, the claustrum’s abundant reciprocal connections with the prefrontal cortex, insula, and cingulate cortex^15^—where slow waves can originate^117–119^—make the claustrum a promising candidate for regulating slow waves. These anatomical connections and physiological findings appear to support a role for the claustrum in sleep-related phenomena. Indeed, the claustrum shows increased expression of markers of neural activity following REM sleep hypersomnia, particularly in claustral neurons connected to frontal cortices such as anterior cingulate cortex.^120^

A recent study by Narikiyo *et al*.^7^ causally linked the claustrum to slow-wave activity. The authors found that stimulation of the claustrum during slow-wave sleep induced a slow wave-like down-state in the cortex followed by a synchronized transition to an up-state (Fig. 3D). In keeping with this, claustrocortical neurons were more active in slow-wave sleep, firing maximally just before and after the up-state of slow waves. Finally, slow-wave activity was reduced by genetic ablation of the claustrum. While this provides a compelling link between the claustrum and sleep, it is important to note that stimulation of the frontal cortex and thalamus^121,122^ as well as peripheral and transcranial magnetic stimulation^123^ have both been reported to elicit similar slow-wave activity (Fig. 3D). As such, further research is required to establish a unique role for the claustrum in slow-wave sleep.

Another recent study by Norimoto *et al*.^115^ linked the claustrum to other aspects of sleep. The authors identified a reptilian homologue of the claustrum that was important in the generation of sharp-wave ripples. These physiological events are composed of a slow, high amplitude potential (sharp wave) followed by a burst of rapid oscillatory activity (ripple) and are implicated in memory consolidation.^124^ Increasing and decreasing serotonin levels in the claustrum led to upregulation and downregulation of spontaneous sharp-wave ripples. They did, however, note that lesions of the claustrum had no effect on the alternation between REM and non-REM sleep. The authors conclude that while the claustrum underlies the generation of sharp-wave ripples during slow-wave sleep, it is not involved in the generation of the sleep rhythm itself.

These studies have carefully dissected several global and specific aspects of sleep and found that while the claustrum was involved with various aspects of sleep in different species, it did not control sleep globally. These results strongly implicate the claustrum as part of a network that subserves some of the neurophysiological correlates of sleep. While the link between the claustrum and sleep remains preliminary, the available evidence from animal studies in combination with the data from human studies presented above might lead to fruitful directions for future research.

### Pain

The lesion studies outlined above hint at the intriguing possibility that the claustrum may play a role in pain perception. Pain is a multifaceted sensation, involving both sensory and affective dimensions. Acutely painful sensations activate, in a flexibly accessible manner, a bilateral and widely distributed network of brain regions. These include a core set of regions, such as the thalamus, insula, brainstem, secondary somatosensory, mid- and anterior-cingulate, and prefrontal cortices (Fig. 3E).^125–127^ Pain-related activity in the claustrum, which is closely connected with many of these brain regions, may be obscured by activity in the nearby insula, falsely attributed to the claustrum when genuinely within the insula, or, if in both, attributed to one or the other.

Figure 3F illustrates the classification results for physical pain within a region of the posterior insula.^126^ It is clear that the extent of activity from the various studies that have contributed to this map might overlap with the adjacent claustrum. Going forward, opportunities with ultra-high field imaging, such as 7 T MRI, with improved signal-to-noise, spatial resolution, and contrast effects are already illustrating for various studies, including pain, the potential it offers for better spatial discrimination between adjacent brain regions or nuclei.^128,129^ It will be important for future studies to better delineate the precise roles of the claustrum and divisions of the insula, as well as their interactions, in pain.

Recent advances in rodent and human imaging now also enable further isolation of claustral and insular activity.^130,131^ Notably, the insula has been identified as a potential deep brain stimulation target for treatment of pain^132,133^; it is possible that electrodes inserted for this purpose also have contacts within the claustrum or affect it via downstream activation. Despite the challenges of isolating claustrum activity during MRI, several studies of human brain activity during various modalities of experimentally induced pain report increased activity in the claustrum, among many other regions.^134–144^

As well as acute pain, claustrum activity has been reported in a few studies looking at chronic pain.^145,146^ Furthermore, studies examining the placebo effect, catastrophizing, and empathy have also reported activity within the claustrum among other regions.^147–152^ The claustrum has further been reported as active in studies investigating acupuncture, migraine, oxygen deprivation, and invasive motor cortex stimulation in patients with chronic pain.^153–157^ Drawing strong conclusions from these studies is challenging as the authors rarely include any discussion of the claustrum outside of data tables.

In addition to human and animal imaging studies, non-imaging evidence from animal studies also supports a link between the claustrum and pain. The claustrum expresses proteins implicated in pain-related signalling, including kappa, delta, and mu opioid receptors,^158–162^ and shows increased neural activity following experimentally-induced pain in various species.^163–167^ One early study assessed the correlation between seizure-induced damage to the brain and changes in rats’ pain threshold. Intriguingly, of all the structures within the diencephalon and telencephalon, damage to the claustrum explained the largest percentage of variance in the pain threshold.^168^ Cautious interpretation is warranted as the pain threshold test in a rat will be confounded with movement and/or motivation deficits.

The claustrum may also play a role in the sensation of itch, a sensation that provokes the desire to scratch. While pain and itch are distinct sensations, the two are closely related.^169–172^ Functional neuroimaging studies have noted differences in baseline claustrum activity in patients with chronic pruritus (itching) and healthy volunteers.^173,174^ Multiple studies have found increased claustrum activity in response to experimentally induced itch,^174–176^ and this activity correlates with itch intensity.^176^ The claustrum is also rich in K-opioid receptors which have been linked to itch reduction.^158,177^ Moreover, claustrum deactivation is positively correlated with itch-reduction following application of butorphanol, a drug with Κ-opioid receptor agonist activity.^178^

Although the claustrum is not commonly reported as active in studies of pain and itch, its potential involvement merits further investigation. The present lack of discussion, possible reporting bias, and inconsistent activation may be due to the dominance of other structures very near the claustrum that have well defined roles in pain and itch. Despite these challenges, and in light of ultra-high field MRI imaging opportunities and animal imaging techniques, further investigation of a connection between the claustrum and pain is warranted—not least because of its accessibility as a target for deep brain stimulation.

## Discussion

Claustrum lesions resulted in numerous symptoms including disturbances in cognitive function, perceptual and motor abilities, mental state, electrical activity, sleep, and pain. Similarly, animal studies have reported roles for the claustrum in numerous functions as outlined above. At first, it seems difficult to reconcile these diverse findings and potential functions. Rather than performing a single function, the claustrum may instead play multiple intersecting roles that underlie the diverse range of disruptions observed following claustrum lesions.

We hypothesise that the widespread connectivity substrate provided by the claustrum may have initially developed for evolutionarily ancient and fundamental functions. Later, it became a useful framework or scaffold upon which more advanced cognitive abilities could be built as they developed over evolutionary timescales. In considering this possibility, it is perhaps helpful to look across the evolutionary history of the claustrum. Putative claustra have been described in all extant mammals, but the claustrum is likely far older. A claustrum homologue was described in birds by Puelles^179^ and more recently a reptilian claustrum was described by Norimoto *et al*.^115^ By comparing more distant branches of the evolutionary tree, we may perhaps extrapolate backwards to the original function of the claustrum. Sleep, one of the most ancient neural processes, has been linked to the claustrum in species separated by hundreds of millions of years of evolution. Norimoto *et al*.^115^ found that the reptilian homologue of the claustrum was involved in the generation of sharp-wave ripples during slow-wave sleep. Similarly, Narikiyo *et al*.^7^ proposed that the claustrum was involved with the generation of slow-wave sleep in mice, and Jansen *et al*.^77^ linked the claustrum to insomnia in humans. While these studies connecting the claustrum with sleep are preliminary (see above), it is clear that the early claustrum was involved in some facet of sleep.

How can this evolutionarily ancient structure involved in the generation of sleep, one of the most ancient of neural processes, also be linked to more complex cognitive processes like salience or sensory processing? While the extensive connectivity of the claustrum may have evolved to facilitate sleep, it may have gained other uses as the brain increased in complexity. With the expansion of the cortex in mammalian lineages, the scaffold of the claustrum linking these diversifying cortices may have taken on new functions to support new behavioural needs. These new functions, by-products of the claustrum’s exceptional connectivity, may have superseded the original function. These functions may even exist as discrete circuits contained within the claustrum complex.

Among the diverse consequences of claustrum lesions, we observed a strikingly high incidence of seizures. Combined with evidence from animal studies linking the claustrum to seizures, these studies are redolent of a role for the claustrum in regulating cortical excitability.^39,40,64^ Further research will be required to understand if this increased risk of seizures is specific to the claustrum, or whether similar results would be obtained from any such large disruption of brain connectivity.

## Conclusion

Recent work on the claustrum is unravelling the multifaceted nature of its role in the brain. We sought to answer the classic question ‘What can’t you do without the claustrum?’ Unfortunately, lesions to the human claustrum are rarely specific due to its anatomy and bilateral location. Such lesions inevitably impact neighbouring areas, resulting in heterogeneous effects ranging from impaired cognitive, perceptual, and motor abilities, to altered electrical activity in the brain, and profound disturbances to mental states and sleep. These diverse findings suggest a far-reaching function for the claustrum.

While recent anatomical findings exclude the claustrum from some functions such as low-level sensory processing (given the lack of inputs from sensory thalamic nuclei and sparse inputs from primary sensory cortices) and direct generation and/or modification of motor output (given lack of connectivity with striatum), research has not yet yielded a conclusive function for the claustrum. It remains possible that the claustrum underlies many discrete functions and/or possesses a, so far, unidentified global function that ties together these disparate findings. The lesion studies described above do not lend conclusive support to any one theory of the claustrum’s function. They do not provide strong evidence for prominent theories such as multisensory integration and salience processing while lending support to other theories such as the regulation of cortical excitability. Further, the lesion studies in the context of current research have led us to hypothesize a role for the claustrum in regulating cortical excitability. While the function—or functions—of the claustrum is not yet known, it is clear that further work is required to understand this enigmatic brain structure.

One possibility is to continue work on the claustrum across species, which has already usefully spanned reptiles, rodents, rabbits, cats, monkeys, and humans. This cross-species approach may be especially important considering that the role of this highly connected brain area could have changed throughout evolution. That the claustrum has been conserved across these diverse lineages over hundreds of millions of years argues for a fundamentally important function.

In neuroscience, we are often tempted to conclude that a brain region or cell type is responsible for a specific function, but this does not always generate understanding. The claustrum is a brain structure where this strategy is particularly unhelpful, urging us to re-think our approach both methodologically and conceptually.

## Supplementary Material

awac114_Supplementary_Data
