# Supplementary material for: Human lesions and animal studies link the claustrum to perception, salience, sleep and pain
Source: Brain. 2022 Mar 28;145(5):1610–23. doi: 10.1093/brain/awac114 (PMC9166552; doi:10.1093/brain/awac114)
Supplement: awac114_Supplementary_Data [file awac114_supplementary_data.pdf]

Appendix - Supplementary information

Study selection: The meta analysis was conducted on Scopus and PubMed by searching "((claustrum) AND (lesion)) OR ((claustrum) AND (injury)) OR ((claustrum) AND (contusion)) OR ((claustrum) AND (trauma))". From this search, 103 studies were found. 5 inclusion and exclusion criteria were used for the study selection in this review. (1) Studies have to be primary sources (clinical) and (2) conducted on humans. (3) The claustrum lesion has to be defined explicitly. (4) The claustrum lesion has to be confirmed with neuroimaging and/or post-mortem techniques. (5) Major lesions and trauma resulting in death were excluded. For case studies, details of the case study have to be given explicitly. Studies written in the English language (unless translated) were included. There was no preference in age or sex in study selection. We have also extended our search to check papers on the first ten pages of Google Scholar by searching "claustrum lesion in humans" and "external capsule lesions in humans" as well as PubMed by searching "(external capsule[Title/Abstract]) AND (lesions)". The extra studies found by this extended search were included if they met the criteria as 'Studies found through extended search'.

Seizure categorisation: The table includes standardised seizure reporting according to ILAE 2017. '*Non-seizure disturbance of consciousness*' used as a general term includes LOC/reduced consciousness not associated with a seizure; consciousness disturbance; electroencephalogram showing a diffuse slow wave of 3–4 Hz; and slight drowsiness.

Seizure and symptom categorisation: 12 main categories were used to summarise the symptoms. The inclusion criteria of each category are below. *(1)Cognitive impairment* includes: deficits in memory, retaining information, learning, concentrating, decision-making and/or disorientation; severe cognitive dysfunction in simple and complex attention, reasoning, problem solving and memory, being unable to recall age and the exact date; consciousness disturbance; cognition gradually became impaired; disorientation with regard to time and place; loss of short-term memory; disorientation in time; abulia; cognitive impairment; bradypsychia; impaired attention and concentration functions and impaired judgement. *(2) Motor disturbance* includes: hemiparesis; was not able to walk without support; hemiplegia. (*3)Visual disturbance* includes: trouble seeing; visual hallucinations; nystagmus; diplopia; persistent bilateral amaurosis; temporary loss of vision. (4) *Auditory disturbance* includes: trouble hearing; auditory hallucinations; hearing loss; tinnitus. (5) *Speech disturbance* includes: any change in language abilities; dysarthria; loss of speech. *(6) Non-seizure EEG abnormality* as results of EEG signals compared to healthy people. *(7) Sleep disturbance* includes: change in sleep quality or quantity; somnolence; hypersomnia; sleep disturbance; restlessness. (8) *Hallucinations* includes: psychosis; experiencing something that is not there; visual hallucinations and reported seeing cats and people who were not present; auditory hallucinations: clicking sounds and strange voices; psychotic behaviour; Cotard delusion. (9) *Delusion* includes: A belief directly contradicted by reality; psychotic behaviour; delusion of infidelity; delusions with paranoid and religious content; repeatedly stating that she was dead. *(10) Tremor* includes: involuntary, rhythmic muscle contraction leading to shaking movements in one or more parts of the body; a tongue tremor of 5-6 Hz; tremors of the finger. *(11) Paraesthesia* includes: abnormal skin sensations including tingling, pricking, chilling, burning, numbness without local cause; abnormal gustatory sensation

Table 1: Details of the case studies that reported claustrum-only lesion studies (n=7)

Study	Hemisphere	Other lesion sites	Pathology	Seizure classification	Observations	Case evolution
Hwang et al. 2014	Unilateral, right	Bilateral external capsule	Unknown	Age - 28<br>Convulsive Status Epilepticus (CSE) (A.I.c Unknown whether focal or generalised)<br>Seizure-free (IV phenytoin, lamotrigine, levetiracetam)<br>EEG showed generalised spikes.<br>Modulation and effect of intervention on EEG not recorded.	**Cognitive impairment** (Confusion, cognitive impairment, short term memory impairments)<br>**Non-seizure EEG abnormality** (Diffuse slow-wave discharges on EEG)<br>**Unilateral non-tremor motor disturbance** (Transient postural instability)<br>**Non-seizure disturbance of consciousness** (Gradual worsening of status epilepticus of unknown aetiology over 3 days without full recovery of consciousness in the latter stages)	Recovery 20 days later after the first seizure
Ishii et al. 2011	Bilateral		Mumps encephalitis	Age - 21<br>Day 1 Unknown onset, motor (tonic-clonic), Convulsive status epilepticus (A.I.c. Unknown whether focal or generalised).<br>Day 14 Focal to bilateral tonic clonic. Periodic discharges on EEG.<br>Day 65 Seizure-free (acute IV Midazolam, intermediate IV midazolam, chronic oral sodium valproate and phenytoin).<br>Location, morphology, time-related features, modulation and effect of intervention on EEG unspecified.	**Cognitive impairment** (confusion and disorientation)<br>**Hallucinations** (visual and auditory)<br>**Visual disturbances** (visual hallucinations)<br>**Auditory disturbances** (auditory hallucinations)<br>**Tremor** (tongue)<br>**Other** (Flu-like symptoms - fever, headache, vomiting)	Complete recovery, control of seizures at day 65 by anticonvulsant (phenytoin and sodium valproate)
Maximov et al. 2018[a]	Left		Ischaemic stroke	Age - 55	**Visual disturbances** (mixed horizontal and rotary nystagmus to the right side)<br>**Auditory disturbances** (decreased hearing)<br>**Paresthesia** (abnormal gustatory sensations on the left-hand side of tongue and peribuccal region, spread to left hand side of face and head)<br>**Other** (Dizziness, static ataxia, and ataxic gait)	4 weeks of therapy elicited full recovery
Mumoli et al. 2014[a]	Bilateral	Bilateral external capsule	Febrile infection-related epilepsy syndrome (FIRES)	Age - 14<br>Unknown onset, motor.<br>Convulsive status epilepticus (CSE) (A.I.b. Focal onset evolving into bilateral convulsive SE).<br>EEG showed focal rhythmic spikes and delta activity with generalisation.<br>Treatment failure (IV valproate and midazolam)<br>Modulation and effect of intervention on EEG not recorded.	**Cognitive impairment** (confusion)<br>**Non-seizure EEG abnormality**<br>**Visual disturbances** (visual disturbances)<br>**Auditory disturbances**<br>**Other** (Fever, generalised seizures, EEG revealed focal rhythmic spikes at 5–10 Hz, auditory disturbances, visual disturbance, refractory development with poor response to drugs.)	Still some seizures
Silva et al. 2018[a]	Bilateral		New-onset refractory status epilepticus (NORSE)	Age - 6<br>Day 1 Unknown onset, convulsive status epilepticus.<br>Seizure free (IV diazepam) but mental status non recovery.<br>Day 2 Seizure free (oral valproate, phenytoin and levetiracetam).<br>"Focal tonic-clonic movements in the left superior limb".	**Non-seizure EEG abnormality** (occipital intermittent rhythmic delta activity)<br>**Other** (Flu-like symptoms, fever, headache)	Complete recovery, control of seizures within a day of anticonvulsants (valproate, phenytoin, and levetiracetam, seizure free for 6 months)

Sperner et al. 1996	Bilateral	Bilateral external capsule	Transient non-viral encephalitis	Age - 12<br>On admission, Convulsive Status Epilepticus (CSE) (A.I.c Unknown whether focal or generalised).<br>Hospitalised, focal-onset impaired awareness.<br>Seizure-free (IV phenytoin and diazepam).<br>EEG showed right-lateralized sharp waves.<br>Modulation and effect of intervention on EEG not recorded.	**Cognitive impairment** (severe cognitive impairment)<br>**Non-seizure EEG abnormality** (EEG displayed generalised slowing and right-sided sharp slow waves for 4 weeks)<br>**Delusions** (psychosis)<br>**Visual disturbances** (temporary loss of vision)<br>**Auditory disturbances** (temporary loss of hearing)<br>**Speech disturbances** (temporary loss of speech)<br>**Unilateral non-tremor motor disturbance** (Not able to walk without support - limb weakness)	Complete recovery
Zuhorn et al. 2020	Bilateral	Bilateral external capsule	Parainfectious autoimmune encephalitis	Age - 54	**Cognitive impairment** (disorientation, stupor, delirious behaviour, mild cognitive impairment)	Complete recovery in neurological and cognitive status. However, the claustrum lesions persisted.

[a] Studies found through extended search
[b] Studies in which individual patient details are not provided; data is presented as aggregate
[c] Studies in foreign language
[d] Poor lesion specificity and so has been excluded from case study table and analysis

Table 2: Details of the case studies that reported claustrum lesion along with other regions (n=31)

Study	Hemisphere	Other lesion sites	Pathology	Seizure classification	Observations	Case evolution
Albayrak et al. 2008	Unilateral, right	Left calcarine cortex, right external capsule	Closed head injury		**Visual disturbances** (Bilateral amaurosis, but pupils isocoric and reactive to light bilaterally. Cortical visual pathways intact.)	Resolution of lesions, but no clinical recovery of vision
Barcikowska et al. 1979[a,c]	Unilateral, side not specified	Lower part of the cortex of the insula, capsula extrema, uncinate and fronto-occipital fascicles	Dejérine-Roussy syndrome		**Unilateral non-tremor motor disturbance** (Transient hemiparesis, thalamic position of hand)<br>**Paresthesia** (hemihypoesthesia, lancinating pains of a hand)	
Biemond 1956[a]	Unilateral, right	3 confluent small foci of softening in the opposite sides of the insula and parietal operculum; area of secondary degeneration in the ventral part of the thalamocortical radiation; marked cellular loss in the posterolateral and posteromedial ventral nuclei of the thalamus. Operative lesion on the right inferior parietal lobe and softening of the medulla	Special type of thalamic syndrome resulting from a slight vascular accident.		**Unilateral non-tremor motor disturbance** (Powerlessness in the left arm and left leg. Hemiparesis on the left side improved over 4 weeks. Heightened achilles tendon reflex on left; reflex of Mayer could not be elicited on left. Abdominal reflexes lower than right. Proprioceptive disturbance in left arm and hand after operation for 3 weeks. Hyperpathia)<br>**Paresthesia** (Gradual increase in pain on left side, cold sensation on left side of abdomen - persisted throughout investigation. Increasing sensitivity to touch - persistent (typical hyperpathia). Could not differentiate the touch of a pin head or point.)<br>**Other** (Depression, suicidal ideation greater left side perspiration)	IV sodium iodide had no change on prognosis. Excision of part of the right posterior parietal cortex. Diminished pain and hypersensitivity, but persisted. Suicide 1.5 years after first observation - gas.
Biemond 1956[a]	Unilateral, right (claustrum not found)	Tumour in region of right external capsule - well defined, no infiltration. Brain distinctly flattened, pressure cone at the tonsils of the cerebellum. Displacement of the insula to the outside and striatum dorsomedially. Internal capsule medially displaced and stretched longitudinally. Compression of the greater part of the right lateral ventricle. Partial demyelination	Necrotic tumour (glioma), suggested origin being claustrum.		**Cognitive impairment** (Bradyphrenia and disorientation)<br>**Unilateral non-tremor motor disturbance** (Minimal paresis of left facial nerve and left arm)<br>**Paresthesia** (Pain localised to the right eye and radiated to the right side of neck - hyperalgesia and hypothermia of the left side of the body.)<br>**Other** (Bilateral papilloedema)	Death after a few months.

		of the putamen and pallidum. External capsule not recognised. Demyelination of corona radiata.				
Chakraborty et al. 2014[a]	Left	Multiple cortical (insular, medial and lateral frontal cortex), and periventricular (caudate head) discrete ring enhancing lesions and associated surrounding oedema	Multiple parenchymal neurocysticercosis		**Sleep disturbances** (Sleep disturbances)<br>**Delusions** (Delusion of jealousy)<br>**Unilateral non-tremor motor disturbance** (left-sided hemiplegia)	Recovered
Chessa et al. 2017	Bilateral	Extensive: white matter of semioval centres, temporal lobe, external capsule, claustrum, subinsular regions and midbrain	Neuropsychiatric systemic lupus erythematosus (NPSLE)		**Cognitive impairment** (Severe cognitive dysfunction in simple and complex attention, reasoning, problem solving and memory, being even unable to recall her age and the exact date.)<br>**Visual disturbances** (Bilateral nystagmus, diplopia)<br>**Speech disturbances** (dysarthria)<br>**Unilateral non-tremor motor disturbance** (lower-limb ataxia and bilateral sixth and left seventh cranial nerve palsies were found. She also had weakness in the right side of her body, lower limbs sensory loss and hyper-reactive deep tendon reflexes with left foot dorsiflexion deficit)	Recovered
Couto et al. 2013	Unilateral, right	Right putamen and external capsule	Right subcortical haemorrhagic stroke		**Unilateral non-tremor motor disturbance** (Left sided hemiparesis and hemianaesthesia)<br>**Other** (No neurological deficits, only complained about pain in left arm, leg and foot)	Recovered after 4 months
Dodgson 1955[a]	Bilateral	Bilateral insular microgyria, abnormal frontal and temporal sulci adjacent to the insula	Neurodevelopmental abnormality		**Other** (Infantile death, significant brain malformation, musculoskeletal abnormalities, cyanosis and stridor breathing)	Died shortly after birth
Hiraga et al. 2014	Bilateral	Bilateral medial temporal lobes	VGK (voltage-gated potassium channel) antibody-associated encephalitis	Age - 65<br>Unknown onset motor (other motor)<br><br>Nature of the intervention and mode of application unspecified.<br>No EEG.	**Cognitive impairment** (Acute consciousness disturbance, comatose with clonic seizures. Memory loss)	Recovered with some memory dysfunction
Ishida et al. 2006 [a,c]	Bilateral	Right hippocampus	Non-herpetic acute limbic encephalitis (NHALE)	Generalised onset, motor (tonic-clonic) seizure; seizure free in response to intervention with undetermined adverse effects (1C).<br><br>Anti-epileptic agents regime not specified. No EEG.	**Cognitive impairment** (Disturbance of short-term memory)<br>**Speech disturbances** (Ataxia)<br>**Unilateral non-tremor motor disturbance** (Ataxia)<br>**Non-seizure disturbance of consciousness** (Consciousness disturbance)<br>**Other** (Change of character)	Resolution of symptoms spontaneously, but short-term memory disturbance persisted

Jellinger et al. 2011	Bilateral	Diffuse tauopathy: parietal, temporal, occipital cortex, frontal cortex, hippocampus, putamen, thalamus and subthalamus, white matter	Tauopathy		**Cognitive impairment** (Progressive decline in spatial orientation and visual functions for 2 years. Preserved memory and motor perception, gradually progressing to dementia, without extrapyramidal signs)<br>**Visual disturbances** (He showed impaired optic fixation, optic ataxia, agraphia, acalculia, ideomotor apraxia, disturbed right–left differentiation but preserved colour matching)	Deceased
Kurokawa et al. 2005[a,c]	Bilateral	Subcortical white matter of insular cortex, external capsule, putamen and globus pallidus	Encephalopathy with unknown cause - sugihiratake mushroom	Focal onset (motor), with progression to generalised convulsive status epilepticus (A.1.a);treatment failure in response to intervention with undetermined adverse effects (1C).<br><br>Anti-epileptic agent regime not specified. No EEG.	**Non-seizure EEG abnormality** (EEG in comatose state showed periodic synchronous discharge (PSD))<br>**Speech disturbances** (Dysarthria)<br>**Unilateral non-tremor motor disturbance** (Weakness and involuntary movements of extremities)	Improvement of seizures gradually
Lapenta et al. 2015[a]	Bilateral	Bilateral hippocampus	New-onset refractory status epilepticus (NORSE)	Age - 17<br>Generalised onset, motor (tonic-clonic). Subsequent Focal motor Status Epilepticus (A.3.a. Repeated focal motor seizures (Jacksonian))<br>Treatment failure (diazepam and valproic acid)<br>Development into Myoclonic Status Epilepticus (prominent epileptic myoclonic jerks) (A.2.a. With coma)<br>Treatment failure (phenytoin, valproic acid, levetiracetam, lacosamide, carbamazepine).<br>EEG showed lateralised (left) seizure onset with periodic discharges and sharp waves.<br><br>Mode of application of intervention unspecified. Modulation and effect of intervention on EEG not recorded.	**Cognitive impairment** (frontal cognitive impairment)<br>**Tremor** (bilateral intention tremor)<br>**Other** (Fever)	When discharged, the patient had bilateral intentional tremor of the hands and myoclonic seizures without loss of consciousness. Slight impairment in frontal functions
Matsuzono et al. 2013	Bilateral	Periventricular	Non-herpetic acute limbic encephalitis		**Cognitive impairment** (impaired cognition)<br>**Non-seizure EEG abnormality** (EEG diffuse wave of 3-4 Hz)<br>**Auditory disturbances** (Hearing loss and tinnitus)<br>**Unilateral non-tremor motor disturbance** (myoclonus)<br>**Tremor** (unstable and wide-based gait - Parkinsonism)<br>**Non-seizure disturbance of consciousness** (Consciousness stupor - GCS 11)	Complete recovery
McKay and Cipolotti 2007[a]	Unilateral, right	Bilateral insula (but less severe changes in left insular cortex),	Herpes simplex encephalitis	Age - 24<br>Focal and motor onset, Convulsive Status Epilepticus (CSE) (A.I.b. Focal onset evolving into bilateral convulsive SE)	**Cognitive impairment** (Short term memory impairment for faces, words and visual information.)<br>**Hallucinations** (musical, tactile and visual)<br>**Cotard** (Cotard delusion)	Complete recovery

		right adjacent white matter		No EEG. No discussion of anti-epileptic medication.	**Delusions** (Cotard delusion)	
McMurtray et al. 2014[a]	Left	Left basal ganglia with adjacent oedema likely affecting the corona radiata and possibly extending to the optic radiations<br>Small periventricular hyperintensities	Haemorrhagic stroke		**Hallucinations** (visual and auditory hallucinations)<br>**Cotard** (hemicotard)<br>**Delusions** (delusions of rotting/decaying of the right (paralysed) side of his body)<br>**Visual disturbances** (visual hallucinations)<br>**Auditory disturbances** (auditory hallucinations)<br>**Unilateral non-tremor motor disturbance** (Right-sided weakness, hemiparesis)	Treated with antipsychotics
Mizutani et al. 2020	Bilateral	Reversible splenial lesion (corpus callosum), also hippocampus, amygdala, insula, perisylvian operculum and basal ganglia	New-onset refractory status epilepticus (NORSE)	Age - 30<br>Generalised onset, motor (tonic-clonic)<br>Drug-resistant ("antiepileptics") Status Epilepticus<br>EEG generalised rhythmic high-voltage slow-background activity, frequent multifocal spikes and sharp waves.<br><br>Nature of the intervention and mode of application unspecified.<br>Modulation and effect of intervention on EEG not recorded.	**No other observations**	Reversible splenial lesion, lasting hyperintensities in claustrum and other regions. Seizures remitted and continued for 1.5 months before improvement
Nixon et al. 2001	Unilateral, right	Bilateral temporal lobe, hippocampi atrophy	Idiopathic	Age - 35<br>Unknown onset, motor<br>Subsequent generalised onset, motor (tonic-clonic). Progression to Convulsive Status Epilepticus (CSE) (A.I.a. Generalised convulsive).<br>Drug resistance with treatment failure (IV phenytoin, acyclovir, phenobarbitone, thiopentone)<br>EEG showed generalised slow-wave activity, progressing to generalised periodic discharges.<br><br>Modulation and effect of intervention on EEG not recorded.	**Cognitive impairment** (confusion, disorientation in time)<br>**Non-seizure disturbance of consciousness** (LOC, slight drowsiness)	Death by cardiovascular and respiratory complications
Nomoto et al. 2007[a]	Bilateral	Bilateral medial temporal lobe, insulae, putamen	Sugihiratake Mushrooms, acute encephalopathy		**Sleep disturbances** (somnolent)<br>**Speech disturbances** (Dysarthria)<br>**Unilateral non-tremor motor disturbance** (mild right hemiparesis including face, tetraplegia)<br>**Non-seizure disturbance of consciousness** (coma)	Recovered
Obara et al. 2008[a]	Bilateral	Bilateral putamen, caudate nuc, nuc accumbens, cysts in cerebral cortex, hindbrain damage too	Sugihiratake / Angel's wing mushroom	Age - 65<br>Unknown onset, motor (tonic-clonic)<br>Convulsive Status Epilepticus (CSE) (A.I.a Generalised convulsive)<br>Seizure free (diazepam and phenytoin)<br><br>Mode of application unspecified. No EEG.	**Speech disturbances** (Dysarthria)<br>**Non-seizure disturbance of consciousness** (Consciousness disturbance, Comatose)	Regained consciousness but could only communicate with simple sentences

Obrador et al. 1957[a,c]	Unilateral, right	Inferoexternal part of the putamen, external capsule	Arterial/vascular lesion		**Unilateral non-tremor motor disturbance** (Hemiparesis in left arm and leg, progression into hemiplegia. 24 hours since first symptoms, relief and movement of left hand restored. Limb - upper and lower - movement restored more slowly.)<br>**Paraesthesia** (Numbness in left lower limb, followed by acute radiation to left upper limb. Paraesthetic sensations months later in the left face and upper limb. Gradual increase in intensity for a couple of months, spread to the entire left side of the body. Hyperalgesia to touch.)<br>**Other** (5 years prodromal arterial hypertension. Dizziness, pain in the right frontotemporal region. Headache rising in severity. Fever persistent for 48 hours. Muscle tone normal, normal neurological limb examination. No cognitive impairment. Normal EEG. Autopsy for claustrum lesion.)	Patient died by suicide following failed surgery to alleviate pain, prodromal depression.
Okamoto et al. 2008[a]	Bilateral	Bilateral medial temporal lobes, insula, basal ganglia, hippocampus	Non-herpetic acute limbic encephalitis (NHALE)	Age - 43<br>Unknown onset, motor (tonic-clinic)<br>Convulsive status epilepticus (CSE) (A.I.c. unknown whether focal or generalised)<br>Treatment failure (“anticonvulsants”).<br>EEG showed periodic sharp waves.<br><br>Nature of the intervention and mode of application unspecified. Location of discharges, modulation and effect of intervention on EEG not recorded.	**Non-seizure EEG abnormality** (EEG showed periodic sharp waves)	Fatal 28 days after onset
Saito et al. 2007[a]	Bilateral	Diffuse cortical atrophy appeared during the recovery phase, decrease in bifrontal blood flow	Bronchopneumonia<br>Acute encephalitis	Age - 10<br>Unknown onset, motor (tonic clonic)<br>Initial drug failure (IV midazolam and barbiturates). Seizure-free (oral phenytoin and clonazepam)<br>EEG showed partial suppression of multifocal spikes with IV high-dose pentobarbital; tapering of dose revealed pattern again. Generalised periodic discharges persisted; IV zonisamide and potassium bromide revealed burst-suppression pattern.	**Non-seizure disturbance of consciousness** (fluctuation of consciousness)<br>**Other** (Headache, fever, EEG and brain appearance normal)	At 60 days, therapeutics resolved fever; seizures persisted. Complete recovery
Seghier et al. 2014	Unilateral, left	All on the left side: putamen, most of the globus pallidus, a dorsal part of caudate nucleus, anterior internal capsule, external capsule, superior occipitofrontal fasciculus and the corona radiata	Stroke in the left lentiform nucleus		**Speech disturbances** (Only aphasic for auditory repetition of words and non-words 15 months after stroke)<br>**Other** (Initial observations not specified)	Not specified

Serrano-Castro et al. 2013[a]	Bilateral	Bilateral insula	Febrile infection-related epilepsy syndrome (FIRES)	Age - 19<br>Focal onset, motor onset, aware<br>Focal motor Status Epilepticus (A.3.a. Repeated focal motor seizures (Jacksonian))<br>EEG showed generalised spikes and sharp waves.<br>Seizure free (IV levetiracetam, clonazepam, methylprednisolone)<br><br>Modulation and effect of intervention on EEG not recorded.	**Speech disturbances** (dysarthria)<br>**Sleep disturbances** (nocturnal seizures)<br>**Other** (Flu-like symptoms, fever)	Recovered
Shiihara et al. 2006	Bilateral	Bilateral hippocampus, amygdala, bilateral mesial temporal	Acute encephalopathy with refractory status epilepticus	Age - 12<br>Focal onset, aware, motor onset.<br>Convulsive Status Epilepticus CSE (Focal onset evolving into bilateral convulsive SE)<br>EEG showed multifocal spikes or sharp waves.<br>Treatment failure with progression to drug resistant CSE (phenobarbital, phenytoin, valproate, midazolam, thiamylal, propofol).<br><br>Mode of application unspecified.<br>Modulation and effect of intervention on EEG not recorded.	**Speech disturbances** (seven months after the onset, she could not speak)<br>**Non-seizure disturbance of consciousness** (Consciousness disturbance without focal neurological signs)	Severe atrophy developed. Seven months after the onset, she could not speak or sit alone.<br>Antiepileptics caused leukopenia, hypotension, atelectasis, thrombophlebitis, renal failure and liver failure
Shintaku et al. 2010	Bilateral	Widespread atrophy and lesions to: cerebral cortex (medial temporal lobe), insular cortex, hippocampus, amygdala, mamillary body, thalamus, pontine base, lumbar cord, cerebellar white matter, midbrain	Human herpes virus 6 (HHV6) induced encephalomyelitis following an allogeneic bone marrow transplant (BMT) to treat acute myeloid leukaemia		**Cognitive impairment** (At 35 days post-BMT: short term memory deficits, space and time disorientation, STM deficit)<br>**Sleep disturbances** (At 35 days post-BMT: somnolence)<br>**Tremor** (At 35 days post-BMT: tremors of the fingers)	4 months after BMT, increased severity of sensory aphasia and tremor.<br>Death after a clinical course of 6 months
Shintani et al. 2000	Unilateral, right	Left thalamus, right temporal lobe, and pons	Gliomatosis cerebri		**Cognitive impairment** (abulia)<br>**Sleep disturbances** (hypersomnia)<br>**Unilateral non-tremor motor disturbance** (Bilateral hemiparesis)	Recovered
Turkalj et al. 2012[a]	Left	A 10 cm tubular area of posttraumatic encephalomalacia of the left hemisphere (left orbitofrontal region, insula, putamen, deep white matter and parietal lobe with consecutively slightly enlarged left lateral	Stabbing injury from a billiard stick		**Cognitive impairment** (bradypsychia anosognosia)<br>**Non-seizure EEG abnormality** (showed slow left temporal activity.)<br>**Hallucinations** (visual hallucinations)<br>**Delusions** (Delusions with paranoid and religious content, Psychosis)<br>**Visual disturbances** (left mydriasis, visual hallucinations)	Recovered from hallucinations

Yoshimura et al. 1988	Not specified, just "claustrum"	Widespread Lewy bodies – diencephalon, brain stem, sympathetic ganglia, entire cerebral cortex	Juvenile Parkinson's		**Cognitive impairment** (Memory loss in later disease)<br>**Sleep disturbances** (restlessness)<br>**Unilateral non-tremor motor disturbance** (weakness of his extremities)<br>**Tremor** (tremor)<br>**Other** (Full signs and symptoms of classical Parkinson's disease – tremor and rigidity of neck and extremities, loss of associated movement, stooped posture, facial masking and pro- and retropulsion. Gait disturbances, stiffness and weakness of extremities, treated well for 4 years with L-DOPA before hospitalisation, hypersexuality)	Progressive worsening of condition until death 14 years after diagnosis.
Yasui et al. 1999[a,c]	Not defined, likely to be unilateral	Hippocampus, amygdala	Post-infectious encephalitis in limbic system after Reye's syndrome	Unknown onset and unclassified status epilepticus; treatment failure in response to intervention with undetermined adverse effects (1C). Anti-epileptic agents regime not specified. No EEG.	**Cognitive impairment** (Limbic dementia)	Improved dramatically but limbic dementia persisted

[a] Studies found through extended search
[b] Studies in which individual patient details are not provided; data is presented as aggregate
[c] Studies in foreign language
[d] Poor lesion specificity and so has been excluded from case study table and analysis

Table 3: Details of the cohort studies that reported claustrum lesion along with other areas (n=14)

Study	Patients (n)	Hemisphere	Other areas	Pathology	Observations	Case evolution
Chau et al. 2015[a,b]	171		Variable and undefined on aggregate; most limited to cortex and white matter	Penetrating head injury	**Non-seizure disturbance of consciousness** (Loss of consciousness (Prolonged N=16, transient N=91, none N=64) "Claustrum damage was associated with the duration, but not frequency, of loss of consciousness, indicating that the claustrum may have an important role in regaining, but not maintaining, consciousness")	
Choi et al. 2019	10/13	Bilateral	3/10 neocortex, 2/10 bilateral pulvinar<br><br>1)Bilateral mesial temporal<br>2) Bilateral mesial temporal<br>3) Bilateral mesial temporal<br>4) Bilateral mesial temporal, multiple neocortex sites and pulvinar<br>6) Bilateral mesial temporal<br>7) Bilateral mesial temporal, multiple neocortex sites and pulvinar<br>10) Bilateral mesial temporal and frontal cortex<br>11) Bilateral mesial temporal<br>12) Bilateral mesial temporal<br>13) Bilateral mesial temporal	New-onset refractory status epilepticus (NORSE)	Prodromal symptoms:<br>1) fever, headache, myalgia, memory impairment<br>2) Fever, upper respiratory tract infection, memory impairment, confusion, Bi-LPD<br>3) Fever, myalgia, headache, memory impairment, language impairment, somnolence, Bi-LPD<br>4) myalgia, upper respiratory tract infection, confusion, GPD<br>6) fever, nausea, memory impairment, GPD<br>7) fever, myalgia, somnolence, confusion, language impairment, Bi-LPD<br>10)headache, confusion, language impairment, GPD<br>11) fever, headache, myalgia, memory impairment, language impairment, Bi-LPD<br>12) fever, headache, myalgia, somnolence, confusion, labile mood, LPD<br>13) fever, myalgia, upper respiratory tract infection, confusion, GPD<br><br>All presented with status epilepticus.<br>GPD = generalised periodic discharge<br>Bi-LPD = bilateral independent lateralised periodic discharge<br>LPD = lateralised periodic discharge	Poor prognosis (with extratemporal/claustrum involvement) BUT scarce data available about the prognostic significance of claustrum involvement.<br><br>Differs between patient.<br>Range:<br>ICU day 4-129<br>Hospital day 26-129
Duffau et al. 2007 [a,b]	42	12 left<br>30 right<br>Complete removal of affected claustrum during surgery	Insula	WHO grade II glioma	All had seizures postoperatively. No description of seizure type and outcome. No EEG.<br><br>No description; mention conspicuous absence of symptoms on CLA lesion	39 patients recovered a normal neurological examination in 3 months<br>3 patients had permanent left hemiparesis (stroke)
Freedman et al. 1984	3	Unilateral, left	Anterolateral to the left frontal horn, anterior portion of the head of the caudate, anterior limb of the internal capsule, anterior putamen, anterior portion of external capsule, insula	Infarction, transcortical motor aphasia (TCMA)	Mild auditory comprehension problems.<br>9) Moderate hemiparesis<br>12) Infarction, facial weakness, stuttering<br>13) Infarction, facial weakness	Not specified
Gustafson et al. 1998[a, d]	2<br>IV:2<br>IV:9	Not specified	Varied and extensive. IV:2 and IV:9 experienced atrophy and degeneration in all following regions, although to different degrees: hypothalamus, corpora mammillaria, central thalamic nuclei, brain stem, substantia nigra, pontine nuclei, cortex	Alzheimer encephalopathy (presenile dementia)	IV:2 and IV:9<br>Generalised motor onset (tonic-clonic) and other motor (myoclonia). No EEG.<br><br>IV:2<br>**Cognitive impairment** (Early amnesia (spatial orientation, but preserved insight) Sypracia, dysgnosia, Logoclonia, Psychomotor slowness)<br>**Delusions** (suspiciousness)	IV:2 death 51yrs after 3 years of condition<br>IV:9 death 56yrs after 7 years of condition

			- temporoparietal, frontal, anterior cingulate, posterior cingulate, sensory motor		**Visual disturbances** (Dyslexia)<br>**Speech disturbances** (Late mutism, Expr and rec dysphasia)<br>**Unilateral non-tremor motor disturbance** (Increased muscular tension, Gait disturbance (bent forwards with long slow steps, “curtseying” at the knees, stiffly and heavily”, Late incontinence)<br>**Other** (Rapid loss of weight)<br><br>IV:9<br>**Cognitive impairment** (Early amnesia (spatial orientation, but preserved insight), Sypracia, dysgnosia)<br>**Sleep disturbances** (Restlessness - agitation)<br>**Delusions** (suspiciousness)<br>**Visual disturbances** (Dyslexia)<br>**Speech disturbances** (Expr and rec dysphasia, Vocally disruptive, Late mutism)<br>**Unilateral non-tremor motor disturbance** (Psychomotor slowness, Increased muscular tension, Gait disturbance, Late incontinence)<br>**Other** (Rapid loss of weight)	
Kim et al. 1997	4	Not specified, “claustrum” only	Bilateral periventricular white matter and centrum semiovale demyelination in all.<br><br>3) Posterior limb of internal capsule, descending pyramidal tracts, cerebellar deep white matter, diffuse corticosubcortical atrophy<br>5) Genu of corpus callosum, splenium of corpus callosum, posterior limb of internal capsule, descending pyramidal tracts, diffuse low T2 intensity in thalami<br>6) Genu of corpus callosum, splenium of corpus callosum, diffuse low T2 intensity in thalami<br>7) Posterior limb of internal capsule, cerebellar deep white matter, diffuse corticosubcortical atrophy, subcortical U fibres.	Late-infantile metachromatic leukodystrophy	All normal developmental processes until onset of symptoms, between 9 and 28 months. Regression of motor development (loss of head control, inability to sit alone, gait disorders, speech disturbances, quadriparesis).	Deceased, not specified.
Leroy et al. 2007[a]	3	Bilateral	Ponto-cerebellar atrophy, cortical changes in insula, decreased brain size	Pontocerebellar hypoplasia (PCH) novel subtype	Fatal seizures in all three cases	Fatal
Meletti et al. 2015	6	Bilateral	1) Insular cortex<br>2) None<br>3) None<br>4) None<br>5)Right posterior thalamus<br>6)None	New-onset refractory status epilepticus (NORSE)	1) Confusion/stupor – staring/eye deviation and focal hemiclonic (uncountable) seizures, then generalised myoclonic state. Status epilepticus evolution was super-refractory.<br>2) Confusion/sleepiness – focal hemiclonic, myoclonic, then generalised (uncountable) seizures. Status epilepticus evolution was refractory.<br>3) Confusion/stupor – focal hemiclonic, myoclonic, then generalised (uncountable) seizures. Status epilepticus evolution was super-refractory.<br>4) Seizures (uncountable) – eye deviation and focal hemiclonic, with alternating side, then generalised. Status epilepticus evolution was refractory.<br>5) Headache/sleepiness – focal hemiclonic, with alternating side, then generalised (uncountable) seizures. Status epilepticus evolution was super-refractory.	1) Normal life, mild deficits in executive functions. Focal epilepsy.<br>2) Cognitive and behavioural deficits. Focal epilepsy.<br>3) Death<br>4) Normal life, slight attentional deficits. Focal epilepsy.<br>5) Normal life.<br>6) Mild cognitive deficit, attention deficit. Seizure-free final follow up.

					6) Stupor/severe sleep disruption – focal motor, tonic, chewing and complex automatisms (isolated) seizures. Status epilepticus evolution was super-refractory.	
Meletti et al. 2017	31	Bilateral	Various	New-onset refractory status epilepticus (NORSE)	For sudden onset altered mental status, ranging from mild fluctuations in mental status to stupor. Status epilepticus was refractory/super-refractory in 74% of the patients, requiring	Differs between patient
Morys et al. 1988[a,b,c]	No detail on which patient has claustrum lesion, 12	Unilateral	Not specified	Not specified	**Other** (Absence of somatosensory evoked potentials contralateral to the side of the lesion and ipsilateral to the stimulated nerve)	
Randerath et al. 2017[a,b]	34	17 Left<br>17 Right		Unilateral infarction or haemorrhagic stroke	**Cognitive impairment** (Anosognosia for motor impairment)<br>**Visual disturbances** (visuo-spatial disturbance)<br>**Unilateral non-tremor motor disturbance** (Motor impairment (limb apraxia), hemiparesis (unaffected ipsilesional hand was used in those patients))<br>**Other** (Neglect symptoms. Assessment of visuo-spatial and motor function indicated right brain damage patients performed worse than those with left-sided damage.)	
Sapir et al. 2007	29	Unilateral	Ventral lateral putamen, white matter beneath the frontal lobe (likely others but not specified for each patient)	Stroke	Hemispatial neglect with directional hypokinesia (individual observations not given)	Not specified
Sener et al. 1998	7	(1) Bilateral<br>(2) Bilateral<br>(3) Bilateral<br>(4) Unilateral, left<br>(5) Unilateral, left<br>(6) Unilateral, left<br>(7) Unilateral, left	(1) Globi pallidi, putamen, head of caudate nuclei<br>(2) Parenchyma<br>(3) Periventricular<br>(4) Left thalamus<br>(5) Left putamen, left caudate nucleus, left opercular region<br>(6) Widespread<br>(7) Widespread	(1) Asphyxia<br>(2) Wilson's disease<br>(3) Ischaemic white matter disease<br>(4) Thalamic arteriovenous malformation<br>(5) MELAS syndrome<br>(6) Viral encephalitis<br>(7) Parkinson's disease	Neurological symptoms are not "attributable to exclusively the claustrum"	Not specified
Snider et al. 2020[a,b]	171	Bilateral peak in functional connectivity	Variable and undefined on aggregate; most limited to cortex and white matter	Penetrating head injury	**Non-seizure disturbance of consciousness** (Loss of consciousness (Prolonged N=16, transient N=91, none N=64) "Suggests that functional anticorrelation with the dorsal brainstem, rather than anatomical intersection with the claustrum, may be the more critical factor, explaining why many penetrating lesions outside the claustrum also cause LOC")	
Steriade et al. 2017	4	Bilateral	(1) Temporal hypersensitivities<br>(2) None<br>(3) None<br>(4) Temporal hypersensitivities	Autoimmune epilepsy	**(1)** Seizures - Generalised motor onset (tonic-clonic). Followed by focal nonmotor onset with impaired awareness. Treatment failure with non-specified antiepileptics and duration of course, and with undetermined adverse effects (2C). Memory impairment and mood disturbance.	(1) Intractable epilepsy<br>(2) Continuation of intermittent seizures

					**(2)** Convulsive SE, unknown whether focal or generalised (A.I.c.). Treatment failure for 12 days with undetermined adverse effects (2C). Two months after discharge, unknown nonmotor onset and, on occasion, generalised motor onset (tonic-clonic) seizures.<br>**(3)** Convulsive SE, unknown whether focal or generalised (A.I.c.) and treatment failure with unknown adverse effects (2C). Followed quickly by NCSE without coma, unknown whether focal or generalised (B.2.c). Patient was initially seizure free (IC) but returned with unknown motor onset seizures; treatment failure with unknown adverse effects (2C).<br>**(4)** Unknown nonmotor onset seizures evolved to convulsive SE, unknown whether focal or generalised onset (A.I.c.). Patient was seizure free with undetermined adverse effects (IC), achieved with unspecified antiepileptics. Withdrawal of treatment saw seizures of unknown nonmotor onset. Cognitive and behavioural disturbance.	(3) Continuation of intermittent seizures<br>(4) Intractable epilepsy and severe cognitive impairment

[a] Studies found through extended search
[b] Studies in which individual patient details are not provided; data is presented as aggregate
[c] Studies in foreign language
[d] Poor lesion specificity and so has been excluded from case study table and analysis

Supplementary Table References

Supplementary Table 1

Hwang KJ, Park KC, Yoon SS, Ahn TB. Unusual lesion in the bilateral external capsule following status epilepticus: a case report. *J Epilepsy Res*. 2014;4(2):88–90.

Ishii K, Tsuji H, Tamaoka A. Mumps virus encephalitis with symmetric claustrum lesions. Am *J Neuroradiol*. 2011;32(7): E139.

Maximov GK, Hinova-Palova DV, Iliev AA, et al. Ischemic stroke of the left claustrum in a 55-year-old female: a case report. *Claustrum*. 2018;3(1):1528135.

Mumoli L, Labate A, Palamara G, Sturniolo M, Gambardella A. Reversible symmetrical external capsule hyperintensity as an early finding of autoimmune encephalitis. *Neurol Sci*. 2014; 35(7):1147–1149.

Silva G, Jacob S, Melo C, Alves D, Costa D. Claustrum sign in a child with refractory status epilepticus after febrile illness: why does it happen? *Acta Neurol Belg.* 2018;118(2):303–305.

Sperner J, Sander B, Lau S, Krude H, Scheffner D. Severe transitory encephalopathy with reversible lesions of the claustrum. *Pediatr Radiol*. 1996;26(11):769–771.

Zuhorn F, Omaimen H, Ruprecht B, et al. Parainfectious encephalitis in COVID-19: 'The Claustrum Sign'. *J Neurol*. 2020; 268(6):2031–2034.

Supplementary Table 2

Albayrak BS, Gorgulu A. Persistent bilateral amaurosis in a child caused by damage to the calcarine cortex and the claustrum in contralateral hemispheres after a closed head injury. J Trauma. 2008;64(6):E81–E82.

Barcikowska M. [Lesions of the insula and operculum: a syndrome]. *Neurol Neurochir Pol.* 1979;13(2):205–209.

Biemond A. The Conduction of pain above the level of the thalamus opticus. *AMA Arch Neurol Psychiatry.* 1956;75(3):231– 244.

Biemond A. The Conduction of pain above the level of the thalamus opticus. *AMA Arch Neurol Psychiatry*. 1956;75(3):231– 244.

Chakraborty S, Singi SR, Pradhan G, Anantha Subramanya H. Neuro-cysticercosis presenting with single delusion: A rare psychiatric manifestation. *Int J Appl Basic Med Res.* 2014;4(2): 131–133.

Chessa E, Piga M, Floris A, Mathieu A, Cauli A. Severe neuropsychiatric systemic lupus erythematosus successfully treated with rituximab: an alternative to standard of care. *Open Access Rheumatol.* 2017;9:167–170.

Couto B, Sedeño L, Sposato LA, et al. Insular networks for emotional processing and social cognition: Comparison of two case reports with either cortical or subcortical involvement. *Cortex*. 2013;49(5):1420-1434. doi:10.1016/j.cortex.2012.08.006

Dodgson MCH. A congenital malformation of insular cortex in man, involving the claustrum and certain subcortical centres. *J Comp Neurol*. 1955;102(2):341-364. doi:10.1002/cne.901020203

Hiraga A, Watanabe O, Kamitsukasa I, Kuwabara S. Voltage-gated Potassium Channel Antibody-associated Encephalitis with Claustrum Lesions. *Intern Med.* 2014;53(19): 2263–2264.

Ishida H, Hattori H, Takaura N, et al. [A child with non-herpetic acute limbic encephalitis affecting the claustrum and hippocampus]. *No Hattatsu Brain Dev*. 2006;38(6):443-447.

Jellinger KA, Grazer A, Petrovic K, et al. Four-repeat tauopathy clinically presenting as posterior cortical atrophy: atypical corticobasal degeneration? *Acta Neuropathol.* 2011;121(2):267–277.

Kurokawa K, Sato H, Nakajima K, Kawanami T, Kato T. [Clinical, neuroimaging and electroencephalographic findings of encephalopathy occuring after the ingestion of “sugihiratake” (Pleurocybella porrigens), an autumn mashroom: a report of two cases]. *Rinsho Shinkeigaku*. 2005;45(2):111-116

Lapenta L, Frisullo G, Vollono C, et al. Super-refractory status epilepticus: Report of a case and review of the literature. *Clin EEG Neurosci.* 2015;46(4):335–339.

Matsuzono K, Kurata T, Deguchi S, et al. Two unique cases with anti-GluR antibody-positive encephalitis. Clin Med Insights Case Rep. 2013;6:113–117.

McKay R, Cipolotti L. Attributional style in a case of Cotard delusion. *Conscious Cogn.* 2007;16(2):349–359.

McMurtray A, Tseng B, Diaz N, Chung J, Mehta B, Saito E. Acute psychosis associated with subcortical stroke: Comparison between basal ganglia and mid-brain lesions. *Case Rep Neurol Med.* 2014; 2014:428425.

Mizutani AU, Shindo A, Arikawa S, et al. Reversible splenial lesion in a patient with new-onset refractory status epilepticus (NORSE). *eNeurologicalSci*. 2020;18:100220. doi:10.1016/j.ensci.2019.100220

Nixon J, Bateman D, Moss T. An MRI and neuropathological study of a case of fatal status epilepticus. *Seizure*. 2001;10(8):588-591. doi:10.1053/seiz.2001.0553

Nomoto T, Seta T, Nomura K, et al. A Case of Reversible Encephalopathy Accompanied by Demyelination Occurring after Ingestion of Sugihiratake Mushrooms. *J Nippon Med Sch*. 2007;74(3):261-264. doi:10.1272/jnms.74.261

Obara K, Wada C, Yoshioka T, Enomoto K, Yagishita S, Toyoshima I. Acute encephalopathy associated with ingestion of a mushroom, Pleurocybella porrigens (angel’s wing), in a patient with chronic renal failure. *Neuropathology*. 2008;28(2):151-156. doi:10.1111/j.1440-1789.2007.00819.x

Obrador S, Dierssen G, Ceballos R. Consideraciones clinicas, neurológicas y anatómicas sobre el llamado dolor talámico. *Acta Neurol Latinoam.* 1957;3:58–77.

Okamoto K, Yamazaki T, Banno H, Sobue G, Yoshida M, Takatama M. Neuropathological Studies of Patients with Possible Non-Herpetic Acute Limbic Encephalitis and So-called Acute Juvenile

Female Non-Herpetic Encephalitis. *Intern Med*. 2008;47(4):231-236. doi:10.2169/internalmedicine.47.0547

Saito Y, Maegaki Y, Okamoto R, et al. Acute encephalitis with refractory, repetitive partial seizures: Case reports of this unusual post-encephalitic epilepsy. *Brain Dev*. 2007;29(3):147-156. doi:10.1016/j.braindev.2006.08.005

Seghier ML, Bagdasaryan J, Jung DE, Price CJ. The importance of premotor cortex for supporting speech production after left capsular-putaminal damage. *J Neurosci.* 2014;34(43):14338–14348.

Shiihara T, Kato M, Ichiyama T, et al. Acute encephalopathy with refractory status epilepticus: Bilateral mesial temporal and claustral lesions, associated with a peripheral marker of oxidative DNA damage. *J Neurol Sci*. 2006;250(1):159-161. doi:10.1016/j.jns.2006.07.002

Shintaku M, Kaneda D, Tada K, Katano H, Sata T. Human herpes virus 6 encephalomyelitis after bone marrow transplantation: Report of an autopsy case. *Neuropathology*. 2010;30(1): 50–55.

Shintani S, Tsuruoka S, Shiigai T. Serial positron emission tomography (PET) in gliomatosis cerebri treated with radiotherapy: a case report. *J Neurol Sci.* 2000;173(1):25–31.

Turkalj I, Stojanovic S, Petrovic K, Njagulj V, Mikov I, Spanovic M. Psychosis following stab brain injury by a billiard stick. *Hippokratia*. 2012;16(3):275–277.

Yoshimura N, Yoshimura I, Asada M, et al. Juvenile Parkinson's disease with widespread Lewy bodies in the brain. *Acta Neuropathol*. 1988;77(2):213–218.

Yasui K, Ieda T, Arahata Y, Suzuki Y, Sobue G. [An adult patient of Reye's syndrome--the possible background mechanism of lesions in claustrum, striatum and limbic system and limbic dementia]. *Rinsho Shinkeigaku*. 1999;39(9):920-924.

Supplementary Table 3

Chau A, Salazar AM, Krueger F, Cristofori I, Grafman J. The effect of claustrum lesions on human consciousness and recovery of function. *Conscious Cogn*. 2015;36:256–264.

Choi JY, Kim EJ, Moon SY, Kim TJ, Huh K. Prognostic significance of subsequent extra-temporal involvement in cryptogenic new onset refractory status epilepticus (NORSE) initially diagnosed with limbic encephalitis. *Epilepsy Res.* 2019;158:106215.

Duffau H, Mandonnet E, Gatignol P, Capelle L. Functional compensation of the claustrum: lessons from low-grade glioma surgery. *J Neurooncol.* 2007;81(3):327–329.

Freedman M, Alexander MP, Naeser MA. Anatomic basis of transcortical motor aphasia. *Neurology*. 1984;34(4):409. doi:10.1212/WNL.34.4.409

Gustafson L, Brun A, Englund E, Hagnell O, Nilsson K, Stensmyr M, Öhlin AK, Abrahamson M. A 50-year perspective of a family with chromosome-14-linked Alzheimer's disease. *Human genetics*. 1998 Mar;102(3):253-7.

Kim TS, Kim IO, Kim WS, et al. MR of childhood metachromatic leukodystrophy. *AJNR Am J Neuroradiol*. 1997;18(4):733-738.

Leroy JG, Lyon G, Fallet C, et al. Congenital pontocerebellar atrophy and telencephalic defects in three siblings: a new subtype. *Acta Neuropathol (Berl)*. 2007;114(4):387-399. doi:10.1007/s00401-007-0248-z

Meletti S, Slonkova J, Mareckova I, et al. Claustrum damage and refractory status epilepticus following febrile illness. *Neurology*. 2015;85(14):1224–1232.

Meletti S, Giovannini G, d'Orsi G. et al. New-onset refractory status epilepticus with claustrum damage: Definition of the clinical and neuroimaging features. Front Neurol. 2017;8:111.

Morys JA, Sloniewski P, Narkiewicz O. Somatosensory evoked potentials following lesions of the claustrum. *Acta Physiologica Polonica*. 1988;39(5-6).

Randerath J, Finkel L, Shigaki C, et al. Does it fit?—Impaired affordance perception after stroke. Neuropsychologia. 2018;108: 92–102.

Sapir A, Kaplan JB, He BJ, Corbetta M. Anatomical correlates of directional hypokinesia in patients with hemispatial neglect. *J Neurosci Off J Soc Neurosci*. 2007;27(15):4045-4051. doi:10.1523/JNEUROSCI.0041-07.2007

Sener RN. Lesions affecting the claustrum. *Comput Med Imaging Graph*. 1998;22(1):57-61. doi:10.1016/S0895-6111(97)00043-8

Snider SB, Hsu J, Darby RR, et al. Cortical lesions causing loss of consciousness are anticorrelated with the dorsal brainstem. Hum Brain Mapp. 2020;41(6):1520–1531.

Steriade C, Tang-Wai DF, Krings T, Wennberg R. Claustrum hyperintensities: a potential clue to autoimmune epilepsy. *Epilepsia open*. 2017 Dec;2(4):476-80.
